# Regulatable In Vivo Biotinylation Expression System in Mouse Embryonic Stem Cells

**DOI:** 10.1371/journal.pone.0063532

**Published:** 2013-05-07

**Authors:** Qin Wang, Ryan T. Wagner, Austin J. Cooney

**Affiliations:** Department of Molecular and Cellular Biology, Baylor College of Medicine, Houston, Texas, United States of America; University of Massachusetts Medical, United States of America

## Abstract

Embryonic stem (ES) cells have several unique attributes, the two most important of which are they can differentiate into all cell types in the body and they can proliferate indefinitely. To study the regulation of these phenomena, we developed a regulatable *in vivo* biotinylation expression system in mouse ES cells. The *E. coli* biotin ligase gene BirA, whose protein product can biotinylate a 15-aa peptide sequence, called the AviTag, was cloned downstream of an IRES. The primary vector containing the doxycycline controlled transactivator gene tTA and IRES-BirA was knocked into the ROSA26 locus by homologous recombination. The secondary vector containing the AviTag tagged hKlf4 gene was exchanged into the ROSA26 locus using Cre recombinase. Western blot analysis showed that the doxycycline induced BirA protein can biotinylate the doxycycline induced AviTag tagged hKlf4 protein. The induction of hKlf4 repressed cell growth in the presence or absence of LIF. Chromatin immunoprecipitation assays using streptavidin beads showed that the AviTag tagged hKlf4 protein could enrich the Nanog enhancer. Our results suggested that the regulatable biotinylation system is promising for the gene function studies in mouse ES cells.

## Introduction

The pluripotency and self-renewal of embryonic stem (ES) cells are largely controlled by core pluripotency factors, Oct4 (Pou5f1, POU family transcription factor), Sox2 (Sox, SRY-related HMG box family transcription factor) and Nanog (NK-2 class homeobox transcription factor) [1]. They are highly expressed in ES cells and repressed during ES cell differentiation. They together bind to the promoters of many genes and activate gene expression to maintain ES cell identity and at the same time repress lineage determinants. Other transcription factors also participate in the regulation of the ES cell pluripotency and self-renewal [2], [3], [4]. LIF (Leukmia inhibitory factor) is essential for the maintenance of mouse ES cells in the undifferentiated state [5]. LIF binds to receptors on the cell membrane and through the parallel signaling pathways of Jak-STAT3-Klf4, MAPK-Tbx3 and PI3K-Tbx3 to activate the expression of pluripotency genes [6].

Kruppel-like factor 4 (Klf4) is a transcription factor that has a C_2_H_2_ zinc finger DNA binding domain at its C terminus. In Drosophila, the Kruppel protein regulates gap formation during embryonic development [7]. In mice, the knockout of Klf4 causes neonatal lethality [8]. In mouse ES cells, Klf4 is highly expressed in the presence of LIF, and is rapidly decreased in the absence of LIF [9], [10]. Klf4 and the core pluripotency factors, Oct4, Sox2 and Nanog, synergistically bind to the promoters of many genes to regulate their expression [11], [12]. Ectopic expression of Oct4, Sox2, Klf4 and c-Myc (OSKM) can reprogram somatic cells to induced pluripotent stem (iPS) cells [13]. In mouse epiblast stem cells (EpiSCs), Klf4 expression is down-regulated, and re-expression of Klf4 can reprogram EpiSCs to the mouse ES cell ground state [14]. In human ES cells, ectopic expression of Klf4 and Klf2 or Klf4 and Oct4 can reprogram human ES cells to the naïve human ES cell state, which is similar to the mouse ES cell state [15]. Knockdown of Klf4 in mouse ES cells has been shown not to cause a substantial morphological change, suggesting that Klf4 function can be somewhat compensated by other Klf family members [10]. In somatic cells, Klf4 has been found to regulate cell proliferation and to be a context dependent oncogene or tumor suppressor gene [16], [17], [18].

A tetracycline inducible expression system in mouse ES cells has been developed [19]. In the absence of doxycycline (Dox, a more stable tetracycline analog), tetracycline transactivator (tTA), which contains the DNA binding domain of Tet Repressor (TetR) and the activation domain of VP16, binds to the Tetracycline Response Element (TRE) on the promoter (containing the TRE and the CMV minimal promoter) and activates transcription. In the presence of Dox, tTA can not bind to the TRE. In this system, tTA is ubiquitously expressed from the ROSA26 locus. This system can tightly control the transgene expression.

Biotin and streptavidin binding is the strongest non-covalent binding in nature. Biotinylation can be carried out *in vitro* and *in vivo* with biotin ligases covalently adding biotin to the lysine residue on a specific target sequence of a protein. An efficient and specific biotinylation system in mammalian cells has been reported [20]: transgene cDNA is tagged with a selected artificial tag, the tagged cDNA and *E.coli* biotin ligase BirA gene are expressed in cells, and the protein product of the tagged cDNA is specifically biotinylated by the BirA protein. On the basis of the tetracycline inducible expression system, we cloned the BirA gene downstream of the IRES, replacing the Venus gene, making the tagged transgene and the BirA gene transcribed from the same mRNA, so that the expression of both the transgene and BirA are regulatable by tetracycline, and that the BirA protein biotinylates the transgene protein in *cis* on the tag. This system combined the tetracycline inducible expression system and the BirA *in vivo* biotinylation system. The hKlf4 gene was introduced into this system as a transgene. We showed that hKlf4 can be induced, biotinylated and functional, and could be used in the affinity purification related studies. The induction of biotinylated hKlf4 strongly repressed cell proliferation and viability with or without LIF. ChIP assays indicated that using streptavidin beads to pull down biotinylated hKlf4 efficiently enriched the Nanog enhancer.

## Materials and Methods

### Cell culture

Wild-type mouse ES (mES) cells [2] were cultured in 37°C and 5% CO_2_ and grown on 0.1% gelatin coated cell culture plates. The mES cells were maintained in mESC medium containing LIF: DMEM, 15% FBS (Atlanta Biologicals), 0.1 mM β-mercaptoethanol, 2 mM L-glutamine, 1x MEM non-essential amino acids, 500 U/ml penicillin/streptomycin and 1000 U/ml LIF (Chemicon). Differentiation of the mES cells was induced by addition of 1 µM retinoic acid (RA) to the mESC medium [21].

### Plasmid construction

The knock-in vector pROSATcHB was derived from the knock-in vector pMWROSATcH [19]. The IRES-BirA was amplified from the plasmid pRceiver-Lv35-hLRH1 (GeneCopoeia) by Platinum *Pfx* DNA polymerase (Invitrogen) with the primers IRESBirA-F: CATGCGACGTCATAGCTCTC, and IRESBirA-R: GAAGATCT *CTA*TTTTTCTGCACTACGCAGG (the introduced BglII site is underlined, and the stop codon is italicized). The pMWROSATcH plasmid was partially digested with both MluI and BglII, and then IRES-BirA was cloned to these sites, producing pROSATcHB, which was confirmed by sequencing.

The exchange vector pPthC-avihKlf4 was derived from pPthC-Oct3/4 [19]. AviTag-hLRH1 was amplified from the plasmid pRceiver-Lv35-hLRH1 (GeneCopoeia). The pPthC-Oct3/4 plasmid was digested with XhoI and NotI, and the SalI-NotI fragment containing AviTag-hLRH1 was ligated to these sites, producing pPthC-avihLRH1FL. The open reading frame (ORF) of human Klf4 (hKlf4) (CCDS6770.2) was amplified from human ES cell cDNAs by *Pfx* DNA polymerase with the primers hKlf4-F: ATTTCGAATAATGAGGCAGCCACCTGG, and hKlf4-R: GGCTCGAG *TTA*AAAATGCCTCTTCATGTG (the BstBI and XhoI sites that were introduced are underlined, and the stop codon is italicized). The hKlf4 ORF was cloned into the BstBI and XhoI sites of the pPthC-avihLRH1FL to fuse with the AviTag at the start codon, producing pPthC-avihKlf4, which was confirmed by sequencing.

### ES cell transfection

10^7^ mES cells were electroporated with the 25 µg AscI linearized knock-in vector pROSATcHB using 230 V and 500 µF. Stable clones were selected by 150-250 ug/ml hygromycin and screened by genomic DNA PCR with the 5′ external primers ROSA26-F: CCTAAAGAAGAGGCTGTGCTTTGG, and EN2SA-R: ACTCCAACCTCCGCAAACTC, and the internal primers GI-F: GTGCTGGTTGTTGTGCTGTC, and Hyg-R: ATAGGTCAGGCTCTCGCTGA. Knock-in clones were confirmed by Southern blot analysis of EcoRV digested genomic DNA hybridized with a 0.4-kb ROSA26 5′ probe [19] or a 1.7-kb IRESBirA internal probe, using QuikHyb Hybridization Solution (Stratagene).

10^5^ knock-in cells were seeded in 6-well plates and cultured overnight, and then transfected with the 2 µg pCAGGSCre vector and 5 µg exchange vector pPthC-avihKlf4 using Lipofectamine 2000 (Invitrogen) [19]. Stable clones were selected using 1.5 µg/ml puromycin in the presence of 0.1 µg/ml Dox (Clontech), and screened by hygromycin sensitivity in the absence of doxycycline and then genomic DNA PCR for confirming occurrence of Cre-mediated recombination. Transfected cells were maintained in the presence of 0.1 µg/ml Dox. For the induction of the transgene expression, after cells were seeded and cultured overnight, cells were washed with DPBS (Invitrogen) two times and cultured in the medium without Dox for two hours, and then the medium was changed with the new medium without Dox.

### Western blot

Cell extracts were run on 10% SDS-PAGE gels and then blotted on PVDF membranes (Millipore). Membranes were blocked in 1xTBS (136 mM NaCl, 20 mM Tris-HCl pH 7.6) containing 5% nonfat milk, incubated with the anti-BirA antibody (Sigma), and then incubated with the HRP-conjugated secondary antibody (Santa Cruz). For detection biotinylated proteins, membranes were blocked in 1xTBS containing 3% BSA (RIA Grade) (USB) and then incubated with the avidin-HRP conjugate (eBioscience). Membranes were washed in 1xTBS containing 0.05% Tween-20. Finally, membranes were detected with the chemiluminescent substrate [21].

### RNA preparation, reverse transcriptase (RT) reaction, and quantitative real-time PCR (qPCR)

RNA was prepared using TRIzol Reagent (Invitrogen). cDNA was generated with oligo(dT)_12–18_ primers and Superscript III reverse transcriptase first strand synthesis kit (Invitrogen). Real-time quantitative PCR was performed using QuantiFast SYBR Green PCR Master Mix (Qiagen) and StepOnePlus Real-Time PCR System (ABI). The expression was normalized to the expression of β-actin [2]. The qPCR primers used in this study are listed in Table S1 [2], [13].

### Chromatin immunoprecipitation (ChIP)

Cells were cross-linked with 1% formaldehyde at room temperature for 10 minutes, and then quenched with 0.125 M glycine. Cells were lysed in the cell lysis buffer (20 mM Tris [pH 8.0], 150 mM NaCl, 2 mM EDTA, 1% TritonX-100, 0.1% SDS), and sonicated into 100–1000 bp DNA fragments. After preclearance with protein A/G beads (Santa Cruz), 10 µl of the supernatant was saved as input, and the rest of the supernatant was incubated with streptavidin beads (Invitrogen) at 4°C. The streptavidin beads were washed with RIPA-like buffer, high salt buffer, LiCl buffer and TE buffer (Millipore). Bound materials were eluted and reverse cross-linked in 1% SDS, 50 mM Tris-HCl (pH 8) and 10 mM EDTA overnight at 65°C. DNA was obtained after RNaseA treatment, protease K treatment, phenol/chloroform extraction, and ethanol precipitation. DNA was analyzed with real-time quantitative PCR, normalized to the input DNA [11]. The qPCR primers are listed in Table S1 [11], [22].

### Statistical analysis

The statistical significance is determined by the Student's two-tailed t test.

## Results

### Establishing the ROSA-TET-BirA system

**Figure 1:**
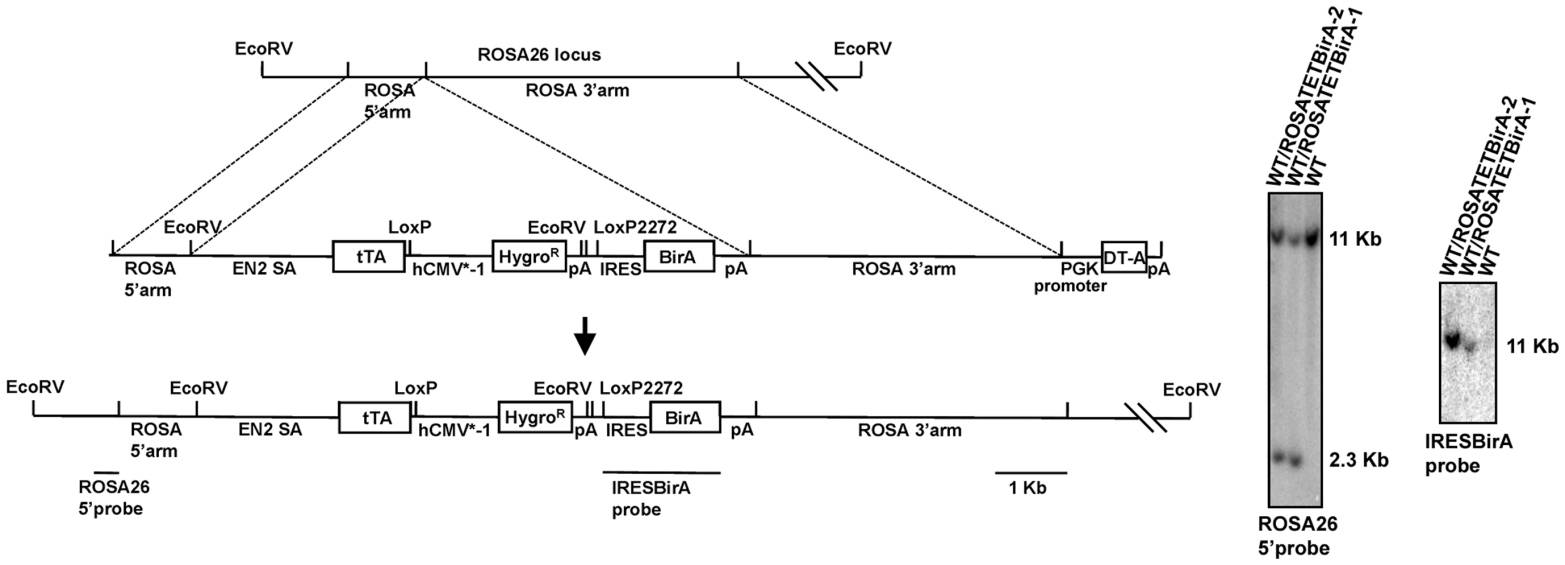
Construction of the knock-in cell lines. The linearized knock-in vector containing the ROSA26 1.2-kb 5′ and 4.2-kb 3′ homology arms was electroporated into the wild-type mES cells to produce recombination with genomic DNA. Genomic DNAs of the wild-type ES cell line and knock-in cell lines were digested with EcoRV and electrophoresed in a 0.8% agarose gel. DNA was hybridized with the ROSA26 5′ probe and IRESBirA probe. The sizes of the hybridization bands are indicated. SA: Splice acceptor; DT-A: Diphtheria toxin A subunit.

Dr. Hitoshi Niwa laboratory established a tetracycline inducible gene expression system knocked into the ROSA26 locus. The vectors utilize modified LoxP sites to facilitate Cre-mediated recombination of cDNAs in a shuttle vector into the ROSA-TET locus for subsequent expression [19]. We wanted to modify the system to combine the powers of this system with *in vivo* biotinylation to allow tagging of the recombinant proteins for subsequent purification applications. The *E. coli* biotin ligase BirA can biotinylate a 15-aa peptide, termed the AviTag. Based on the tetracycline inducible system in mES cells [19], we introduced BirA gene into this system in place of the original Venus gene in the ROSA26 locus. First, we cloned the BirA gene downstream of the IRES in the knock-in vector, replacing the original Venus gene. Next, by homologous recombination, the knock-in vector was targeted into the ROSA26 locus between exon I and exon II in mES cells. The correct targeting events were selected by hygromycin resistance, screened by genomic DNA PCR, and confirmed by Southern blot analysis (Figure 1). Two homologous recombination clones were obtained from 12 hygromycin resistance clones. Using the RMCE (Recombinase-Mediated Cassette Exchange) strategy, in the knock-in cell line, we co-transfected the Cre recombinase expression vector and the exchange vector containing the AviTag tagged cDNA (hKlf4). After puromycin selection in the presence of Dox, stable clones were screened by hygromycin sensitivity in the absence of Dox. Site-specific recombination clones were confirmed by genomic DNA PCR.

### The hKlf4 ORF was introduced into this system

**Figure 2:**
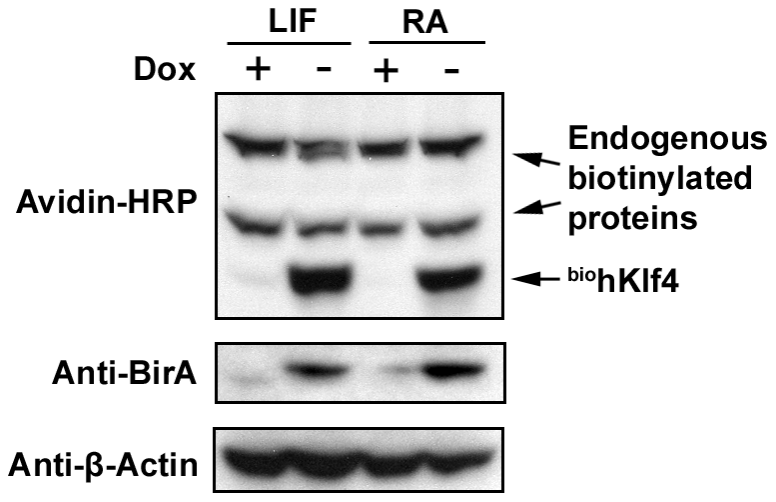
The BirA protein and biotinylated hKlf4 protein were induced in the absence of Dox. The inducible hKlf4 mES cells were cultured in the presence of LIF or RA for 1.5 days. Western blot was performed with cell extracts.

The amino acid sequences of hKlf4 and mKlf4 have 91% similarity by BLAST search, with 100% identity in their zinc finger DNA binding domains. Expression of hKlf4 in the mES cells was induced in the absence of Dox under two different conditions, one in the undifferentiated state maintained by LIF and the second in the RA induced differentiation condition treated for 1.5 days. The protein expression in cell extracts was detected by Western blot analysis (Figure 2). The avidin-HRP conjugate detected strong bands at the size corresponding to the biotinylated hKlf4 protein in the absence of Dox in both the LIF condition and the RA condition. Also, in all the samples, the avidin-HRP conjugate detected endogenous biotinylated proteins, the expression levels of which were lower than those of the induced biotinylated hKlf4 proteins. Western blot analysis using the anti-BirA antibody detected the BirA bands in the absence of Dox in both the undifferentiated LIF condition and the differentiated RA condition. When Dox was present, the biotinylated hKlf4 protein and the BirA protein were almost undetectable, indicating that this regulatable *in vivo* biotinylation expression system was relatively tight.

**Figure 3:**
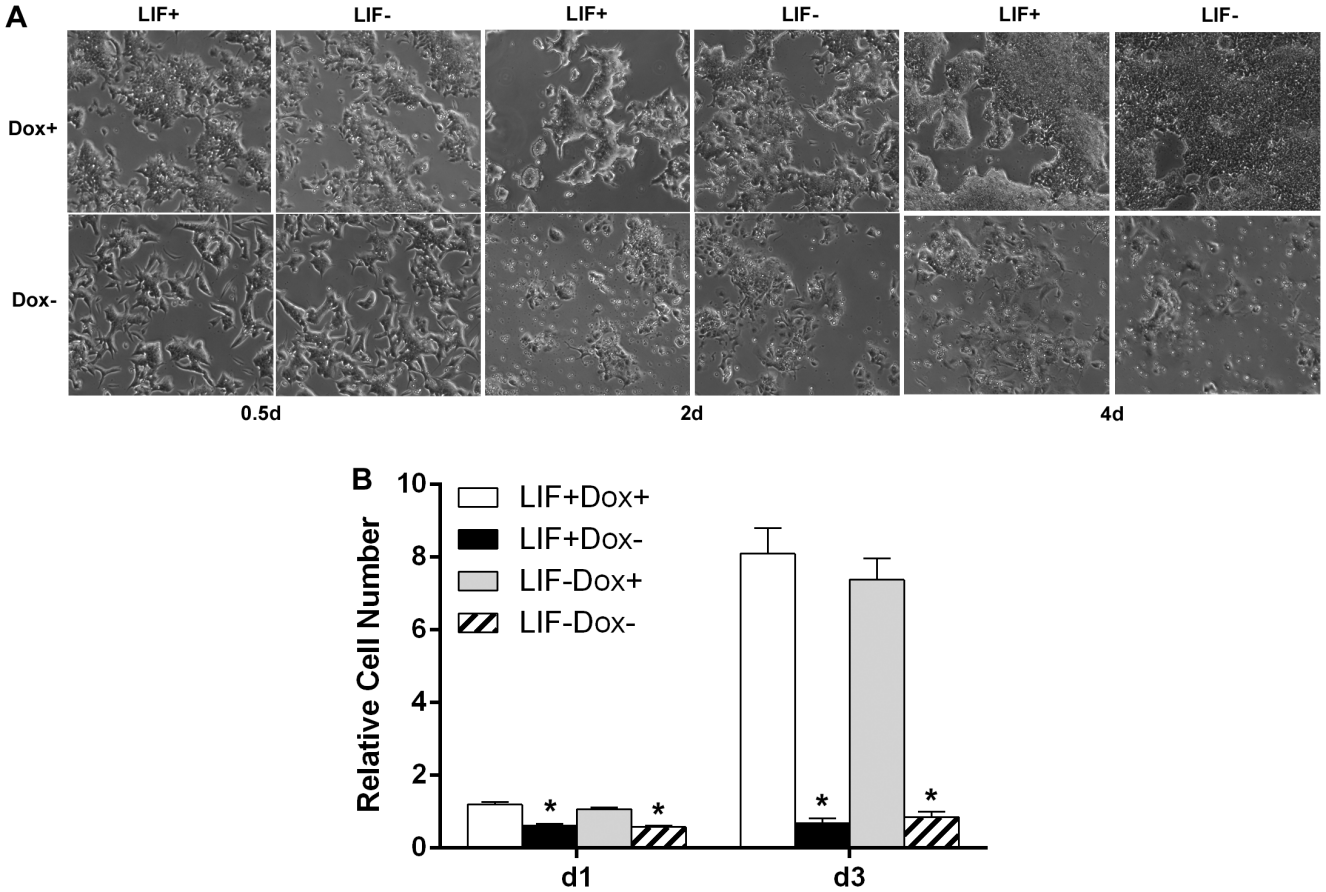
Effects of hKlf4 induction on mES cell growth. The inducible hKlf4 mES cells were cultured in the presence or absence of LIF. (A) Microscopy was carried out with the Zeiss Axio Observer A1 inverted microscope and an AxioCam MRM camera (Bright field, 5x objective lens). (B) Cells were seeded in triplicate wells at 3×10^4^ cells per well of 24-well plates. After induction for one day and three days, cells were trypsinized and counted with a coulter counter (Beckman Coulter Z1 Particle Counter). The cell numbers were plotted relative to the cell numbers of the LIF+Dox+ at day one. Bars represent the average±SEM of two independent experiments with triplicates each. LIF+Dox− was compared with LIF+Dox+, and LIF−Dox− was compared with LIF−Dox+ for statistical significance. *, *p*<0.05.

It has been reported that Klf4 expression is regulated by the LIF signaling pathway [6], [23], thus the hKlf4 ES cell line was cultured in the presence or absence of LIF (Figure 3A). As expected, in the presence of LIF, cells showed compact and undifferentiated morphology. In the absence of LIF, cells grew more flattened. After inducing forced expression of hKlf4, within 12 hours, obvious differences in cell morphology were observed compared with the un-induced control. After two days of induction, the ES cells expressing hKlf4 grew less than the control cells. After four days of induction, the ES cells expressing hKlf4 grew much less than the cells without induction of hKlf4. Cell proliferation assays showed that after one day of induction the cell numbers of the hKlf4 expressing cells were around two-fold lower than those of the Dox+ control cells, and after three days of induction the cell numbers of the hKlf4 expressing cells were around 10-fold fewer than those of the Dox+ control cells (Figure 3B). Therefore, hKlf4 induction strongly affected cell growth and viability in the presence or absence of LIF.

**Figure 4:**
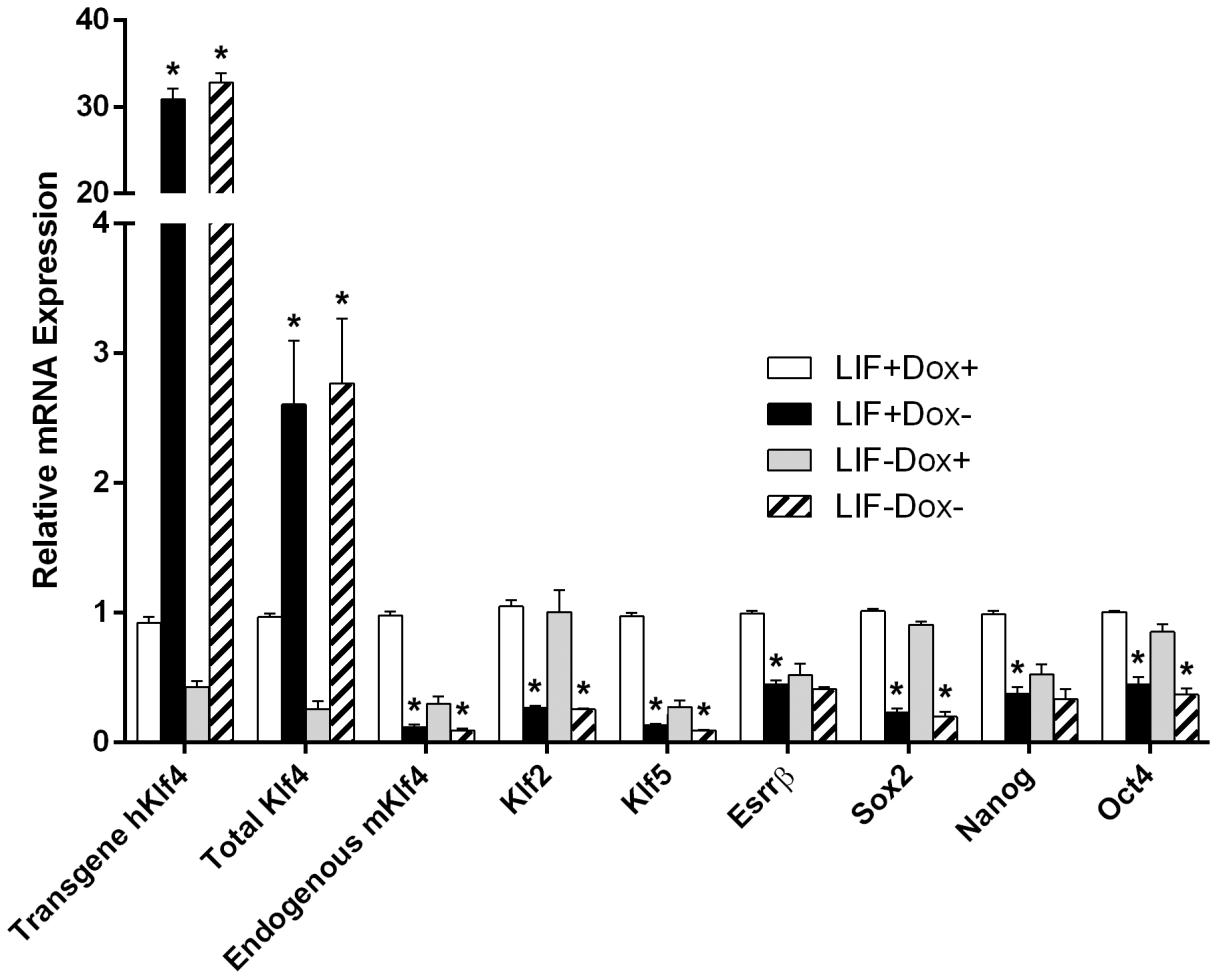
Gene expression effects of hKlf4 induction. The inducible hKlf4 mES cells were cultured with or without LIF for 1.5 days. Real-time RT-qPCR quantified the relative mRNA levels. Bars represent the mean±SEM of at least two independent experiments with two repeats each. LIF+Dox− was compared with LIF+Dox+, and LIF−Dox− was compared with LIF−Dox+ for statistical significance. *, *p*<0.05.

The effects of expression of hKlf4 in mES cells on gene expression were analyzed. Gene expression was quantified by real-time RT-qPCR (Figure 4). Cells were harvested after induction for 1.5 days. Recombinant hKlf4 mRNA expression was detected using specific hKlf4 qPCR primers. In the absence of Dox, hKlf4 mRNA was highly induced, and in contrast, in the presence of Dox, hKlf4 mRNA was almost undetectable. Total Klf4 mRNA, including transgene hKlf4 mRNA and endogenous mKlf4 mRNA, was detected by specific conserved total Klf4 primers. As expected, in the control Dox+ ES cells, total Klf4 was expressed at high levels in the presence of LIF, and expressed at low levels in the absence of LIF. In the Dox− hKlf4 expressing ES cells, total Klf4 was expressed significantly higher than total Klf4 in the Dox+ control cells. In addition, endogenous mKlf4 mRNA was detected by specific mKlf4 primers. In the Dox− hKlf4 expressing ES cells, endogenous mKlf4 was expressed significantly lower than in the Dox+ control cells, suggesting that endogenous mKlf4 was repressed by the induction of hKlf4, and that endogenous mKlf4 was auto-regulated to prevent its overexpression.

Several Klf family members, Klf2, Klf4 and Klf5, are expressed in mES cells and down-regulated during mES cell differentiation [10], [24]. Klf2, Klf4 and Klf5 cooperatively bind to target genes and regulate their expression [10]. Klf2 and Klf5 can replace Klf4 in OSKM mediated reprogramming of somatic cells to iPS cells [25]. In the Dox− hKlf4 expressing ES cells, Klf2 and Klf5 were expressed significantly lower than those in the Dox+ control cells, suggesting that they were repressed by the induction of hKlf4. It has been demonstrated that Esrrβ can replace Klf4 in OSKM mediated somatic cell reprogramming [25]. Esrrβ and Klf4 have many common targets [25]. Klf2, Klf4 and Klf5 target Esrrβ, and Esrrβ targets Klf4 and Klf5, indicating they are mutually regulated [25]. Likewise, in the LIF+Dox− hKlf4 expressing ES cells, Esrrβ was expressed significantly lower compared with Esrrβ in the LIF+Dox+ control ES cells. Oct4, Sox2 and Nanog are core pluripotency factors. They are highly expressed in ES cells and decreased during differentiation. In the Dox− hKlf4 expressing ES cells, in the presence or absence of LIF, Sox2 was significantly repressed by the induced hKlf4. In the LIF+Dox− hKlf4 expressing ES cells, Nanog was expressed significantly lower compared with Nanog in the LIF+Dox+ control ES cells. Also, in the Dox− hKlf4 expressing ES cells, in the presence or absence of LIF, Oct4 was significantly repressed by hKlf4 induction, repression levels of which were less than those of Sox2.

**Figure 5:**
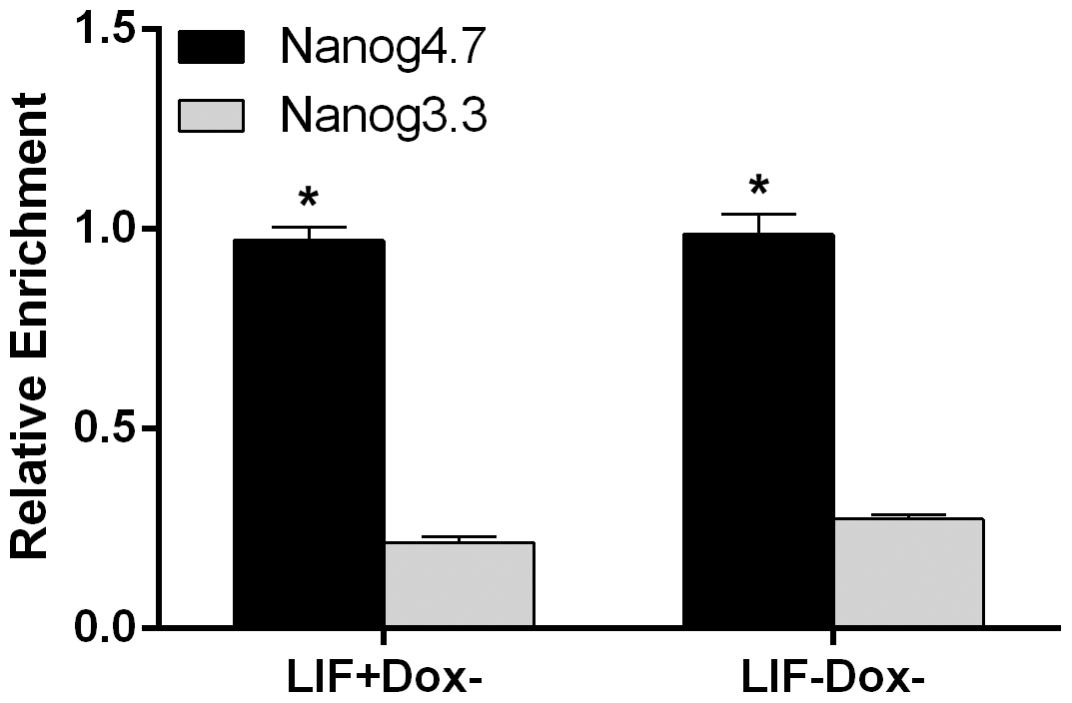
ChIP analysis using streptavidin beads of the hKlf4 expressing mES cells. The cells were cultured with or without LIF in the absence of Dox for 1.5 days. The Nanog upstream 4.7 kb enhancer (Nanog4.7) was enriched by the induction of biotinylated hKlf4. The Nanog upstream 3.3 kb region (Nanog3.3) was used as a control. Bars represent the average±SEM of at least two independent experiments with two repeats each. *, *p*<0.05.

To detect whether regulatable biotinylated hKlf4 could be used with streptavidin beads in ChIP assays, we tested a known Klf4 target, the Nanog upstream enhancer located 4.7 kb upstream of the transcription start site (TSS) [10], [11], [22]. After induction for 1.5 days, cells were cross-linked and sonicated, and the extracts were then incubated with streptavidin beads. DNA enrichment was detected with specific primers (Figure 5). Results showed that in the Dox− hKlf4 expressing cells, in the presence or absence of LIF, the Nanog upstream enhancer was significantly enriched, compared with the control region located 3.3 kb upstream of the Nanog TSS.

## Discussion

We have constructed a new regulatable *in vivo* biotinylation system for mES cells. The biotin ligase gene BirA was cloned downstream of the IRES. Both the BirA and the recombinant cDNA, in this case hKlf4, tagged with the AviTag were transcribed from the same transcript. The expression cassette was integrated at the ROSA26 locus (Figure 1), a relatively ubiquitous and moderate expression locus, which greatly reduces the variable effects caused by random integration in the genome. In the Dox− induced ES cells, the biotin ligase BirA efficiently biotinylated the AviTag (Figure 2). Biotin and streptavidin have the strongest binding affinity in nature. Tagging proteins with biotin reduces the dependence on specific antibodies. A Dox regulatable system is very useful for the protein expression, such as for the expression of proteins toxic to the cells. We found that the RMCE efficiency is low in the BirA system, 1–10%, compared with that in the Venus system [19].

Using the regulatable *in vivo* biotinylation expression system in mES cells, we showed that hKlf4 was induced, biotinylated and functional. High-level hKlf4 induction in the presence or absence of LIF reduced cell proliferation and viability (Figure 3), indicating that hKlf4 played a very important role in regulating mES cell growth and self-renewal. This is supportive of a previous report that Klf4 overexpression is toxic to mES cells [6]. In contrast, when we similarly induced several other genes, no obvious morphological changes were observed (data not shown). The role of Klf4 in cell growth has been well studied as a proliferation inhibitor. In NIH 3T3 cells, Klf4 is highly expressed in quiescent cells compared with proliferating cells [16]. Transcript profiling with inducible Klf4 expression in RKO cells shows that Klf4 globally represses expression of genes involved in promoting the cell cycle, protein biosynthesis, transcription and cholesterol biosynthesis [26]. Our research showed that hKlf4 induction significantly regulated the expression of many genes (Figure 4). The hKlf4 induction repressed endogenous mKlf4, Klf2, Klf5 and the closely related Esrrβ. The expression pattern of endogenous mKlf4 was very similar to that of Klf5. It has been reported that Klf5 often acts as a proliferation enhancer [27]. In mES cells, the targets of Klf5 overlap with those of Klf4, but have distinct differences [10], [28]. How Klf4 and Klf5 work cooperatively in mES cells remains elusive. The Esrrβ expression pattern was very similar to that of Nanog, consistent with the finding that Esrrβ regulates Nanog, and that the mosaic cells expressing Esrrβ correlate with those expressing Nanog [29]. Among Oct4, Sox2 and Nanog core pluripotency factors, Sox2 was most dramatically repressed by hKlf4 induction. Also, we found by RT-qPCR that the pluripotency factors Gdf3, Nodal, Rex1 and Tbx3 and the cell cycle regulator p53 were repressed by the induction of hKlf4 (Figure S1). It has been reported that p53 could be down-regulated by Klf4 in tumor cells [17]. The previous findings showed that Klf4 is downstream of the LIF pathway and contributes to ES cell pluripotency [6], [30], [31]. We also observed that when Klf4 was induced at low levels, pluripotency factors could be activated in the absence of LIF (data not shown). Overexpression of several pluripotency genes, such as Oct4, Sox2 and Tbx3, has been reported to repress the expression of pluripotency genes and activate the expression of the lineage marker genes [32], [33], [34]. At higher expression levels, Klf4 may interact with different transcription factors to repress the target gene expression. In different cellular contexts, Klf4 may differentially regulate gene expression.

Regulatable *in vivo* biotinylated hKlf4 can be used for ChIP assays with pull-down using streptavidin beads (Figure 5). It has been reported that Klf4 directly binds to the Nanog upstream enhancer [10], [11], [22]. In the Dox− hKlf4 expressing ES cells, in the presence or absence of LIF, Nanog enhancer was greatly enriched, consistent with the induced expression of hKlf4. This suggests that at the initial step of ES cell differentiation, Nanog could be repressed when hKlf4 bound to the promoter. This system may provide a powerful approach for the study of gene regulation mechanisms in ES cells.

## Supporting Information

Figure S1 **Gene expression effects of hKlf4 induction were detected by real-time RT-qPCR.**
(TIF)

Table S1 **Primers used for quantitative real-time PCR.**
(DOC)

## References

[1] Niwa H (2007) How is pluripotency determined and maintained? Development 134: 635–646. 17215298 10.1242/dev.02787

[2] Wagner RT, Xu X, Yi F, Merrill BJ, Cooney AJ (2010) Canonical Wnt/beta-catenin regulation of liver receptor homolog-1 mediates pluripotency gene expression. Stem Cells 28: 1794–1804. 20734354 10.1002/stem.502 PMC2996860

[3] Gu P, Goodwin B, Chung AC, Xu X, Wheeler DA, et al (2005) Orphan nuclear receptor LRH-1 is required to maintain Oct4 expression at the epiblast stage of embryonic development. Mol Cell Biol 25: 3492–3505. 15831456 10.1128/MCB.25.9.3492-3505.2005 PMC1084298

[4] Young RA (2011) Control of the embryonic stem cell state. Cell 144: 940–954. 21414485 10.1016/j.cell.2011.01.032 PMC3099475

[5] Smith AG, Heath JK, Donaldson DD, Wong GG, Moreau J, et al (1988) Inhibition of pluripotential embryonic stem cell differentiation by purified polypeptides. Nature 336: 688–690. 3143917 10.1038/336688a0

[6] Niwa H, Ogawa K, Shimosato D, Adachi K (2009) A parallel circuit of LIF signalling pathways maintains pluripotency of mouse ES cells. Nature 460: 118–122. 19571885 10.1038/nature08113

[7] Nusslein-Volhard C, Wieschaus E (1980) Mutations affecting segment number and polarity in Drosophila. Nature 287: 795–801. 6776413 10.1038/287795a0

[8] Segre JA, Bauer C, Fuchs E (1999) Klf4 is a transcription factor required for establishing the barrier function of the skin. Nat Genet 22: 356–360. 10431239 10.1038/11926

[9] Schulz H, Kolde R, Adler P, Aksoy I, Anastassiadis K, et al (2009) The FunGenES database: a genomics resource for mouse embryonic stem cell differentiation. PLoS One 4: e6804. 19727443 10.1371/journal.pone.0006804 PMC2731164

[10] Jiang J, Chan YS, Loh YH, Cai J, Tong GQ, et al (2008) A core Klf circuitry regulates self-renewal of embryonic stem cells. Nat Cell Biol 10: 353–360. 18264089 10.1038/ncb1698

[11] Kim J, Chu J, Shen X, Wang J, Orkin SH (2008) An extended transcriptional network for pluripotency of embryonic stem cells. Cell 132: 1049–1061. 18358816 10.1016/j.cell.2008.02.039 PMC3837340

[12] Chen X, Xu H, Yuan P, Fang F, Huss M, et al (2008) Integration of external signaling pathways with the core transcriptional network in embryonic stem cells. Cell 133: 1106–1117. 18555785 10.1016/j.cell.2008.04.043

[13] Takahashi K, Yamanaka S (2006) Induction of pluripotent stem cells from mouse embryonic and adult fibroblast cultures by defined factors. Cell 126: 663–676. 16904174 10.1016/j.cell.2006.07.024

[14] Guo G, Yang J, Nichols J, Hall JS, Eyres I, et al (2009) Klf4 reverts developmentally programmed restriction of ground state pluripotency. Development 136: 1063–1069. 19224983 10.1242/dev.030957 PMC2685927

[15] Hanna J, Cheng AW, Saha K, Kim J, Lengner CJ, et al (2010) Human embryonic stem cells with biological and epigenetic characteristics similar to those of mouse ESCs. Proc Natl Acad Sci U S A 107: 9222–9227. 20442331 10.1073/pnas.1004584107 PMC2889088

[16] Shields JM, Christy RJ, Yang VW (1996) Identification and characterization of a gene encoding a gut-enriched Kruppel-like factor expressed during growth arrest. J Biol Chem 271: 20009–20017. 8702718 10.1074/jbc.271.33.20009 PMC2330254

[17] Rowland BD, Bernards R, Peeper DS (2005) The KLF4 tumour suppressor is a transcriptional repressor of p53 that acts as a context-dependent oncogene. Nat Cell Biol 7: 1074–1082. 16244670 10.1038/ncb1314

[18] McConnell BB, Ghaleb AM, Nandan MO, Yang VW (2007) The diverse functions of Kruppel-like factors 4 and 5 in epithelial biology and pathobiology. Bioessays 29: 549–557. 17508399 10.1002/bies.20581 PMC2211634

[19] Masui S, Shimosato D, Toyooka Y, Yagi R, Takahashi K, et al (2005) An efficient system to establish multiple embryonic stem cell lines carrying an inducible expression unit. Nucleic Acids Res 33: e43. 15741176 10.1093/nar/gni043 PMC552969

[20] de Boer E, Rodriguez P, Bonte E, Krijgsveld J, Katsantoni E, et al (2003) Efficient biotinylation and single-step purification of tagged transcription factors in mammalian cells and transgenic mice. Proc Natl Acad Sci U S A 100: 7480–7485. 12802011 10.1073/pnas.1332608100 PMC164612

[21] Gu P, LeMenuet D, Chung AC, Mancini M, Wheeler DA, et al (2005) Orphan nuclear receptor GCNF is required for the repression of pluripotency genes during retinoic acid-induced embryonic stem cell differentiation. Mol Cell Biol 25: 8507–8519. 16166633 10.1128/MCB.25.19.8507-8519.2005 PMC1265758

[22] Zhang P, Andrianakos R, Yang Y, Liu C, Lu W (2010) Kruppel-like factor 4 (Klf4) prevents embryonic stem (ES) cell differentiation by regulating Nanog gene expression. J Biol Chem 285: 9180–9189. 20071344 10.1074/jbc.M109.077958 PMC2838337

[23] Hall J, Guo G, Wray J, Eyres I, Nichols J, et al (2009) Oct4 and LIF/Stat3 additively induce Kruppel factors to sustain embryonic stem cell self-renewal. Cell Stem Cell 5: 597–609. 19951688 10.1016/j.stem.2009.11.003

[24] Bruce SJ, Gardiner BB, Burke LJ, Gongora MM, Grimmond SM, et al (2007) Dynamic transcription programs during ES cell differentiation towards mesoderm in serum versus serum-freeBMP4 culture. BMC Genomics 8: 365. 17925037 10.1186/1471-2164-8-365 PMC2204012

[25] Feng B, Jiang J, Kraus P, Ng JH, Heng JC, et al (2009) Reprogramming of fibroblasts into induced pluripotent stem cells with orphan nuclear receptor Esrrb. Nat Cell Biol 11: 197–203. 19136965 10.1038/ncb1827

[26] Whitney EM, Ghaleb AM, Chen X, Yang VW (2006) Transcriptional profiling of the cell cycle checkpoint gene kruppel-like factor 4 reveals a global inhibitory function in macromolecular biosynthesis. Gene Expr 13: 85–96. 17017123 10.3727/000000006783991908 PMC1626270

[27] Ghaleb AM, Nandan MO, Chanchevalap S, Dalton WB, Hisamuddin IM, et al (2005) Kruppel-like factors 4 and 5: the yin and yang regulators of cellular proliferation. Cell Res 15: 92–96. 15740636 10.1038/sj.cr.7290271 PMC1317089

[28] Parisi S, Cozzuto L, Tarantino C, Passaro F, Ciriello S, et al (2010) Direct targets of Klf5 transcription factor contribute to the maintenance of mouse embryonic stem cell undifferentiated state. BMC Biol 8: 128. 20875108 10.1186/1741-7007-8-128 PMC2955566

[29] van den Berg DL, Zhang W, Yates A, Engelen E, Takacs K, et al (2008) Estrogen-related receptor beta interacts with Oct4 to positively regulate Nanog gene expression. Mol Cell Biol 28: 5986–5995. 18662995 10.1128/MCB.00301-08 PMC2547019

[30] Li Y, McClintick J, Zhong L, Edenberg HJ, Yoder MC, et al (2005) Murine embryonic stem cell differentiation is promoted by SOCS-3 and inhibited by the zinc finger transcription factor Klf4. Blood 105: 635–637. 15358627 10.1182/blood-2004-07-2681

[31] Bourillot PY, Aksoy I, Schreiber V, Wianny F, Schulz H, et al (2009) Novel STAT3 target genes exert distinct roles in the inhibition of mesoderm and endoderm differentiation in cooperation with Nanog. Stem Cells 27: 1760–1771. 19544440 10.1002/stem.110

[32] Niwa H, Miyazaki J, Smith AG (2000) Quantitative expression of Oct-3/4 defines differentiation, dedifferentiation or self-renewal of ES cells. Nat Genet 24: 372–376. 10742100 10.1038/74199

[33] Lu R, Yang A, Jin Y (2011) Dual functions of T-box 3 (Tbx3) in the control of self-renewal and extraembryonic endoderm differentiation in mouse embryonic stem cells. J Biol Chem 286: 8425–8436. 21189255 10.1074/jbc.M110.202150 PMC3048727

[34] Kopp JL, Ormsbee BD, Desler M, Rizzino A (2008) Small increases in the level of Sox2 trigger the differentiation of mouse embryonic stem cells. Stem Cells 26: 903–911. 18238855 10.1634/stemcells.2007-0951

