# Supplementary figures and images for: Regulatable In Vivo Biotinylation Expression System in Mouse Embryonic Stem Cells
Source: PLoS One. 2013 May 7;8(5):e63532. doi: 10.1371/journal.pone.0063532 (PMC3646753; doi:10.1371/journal.pone.0063532)

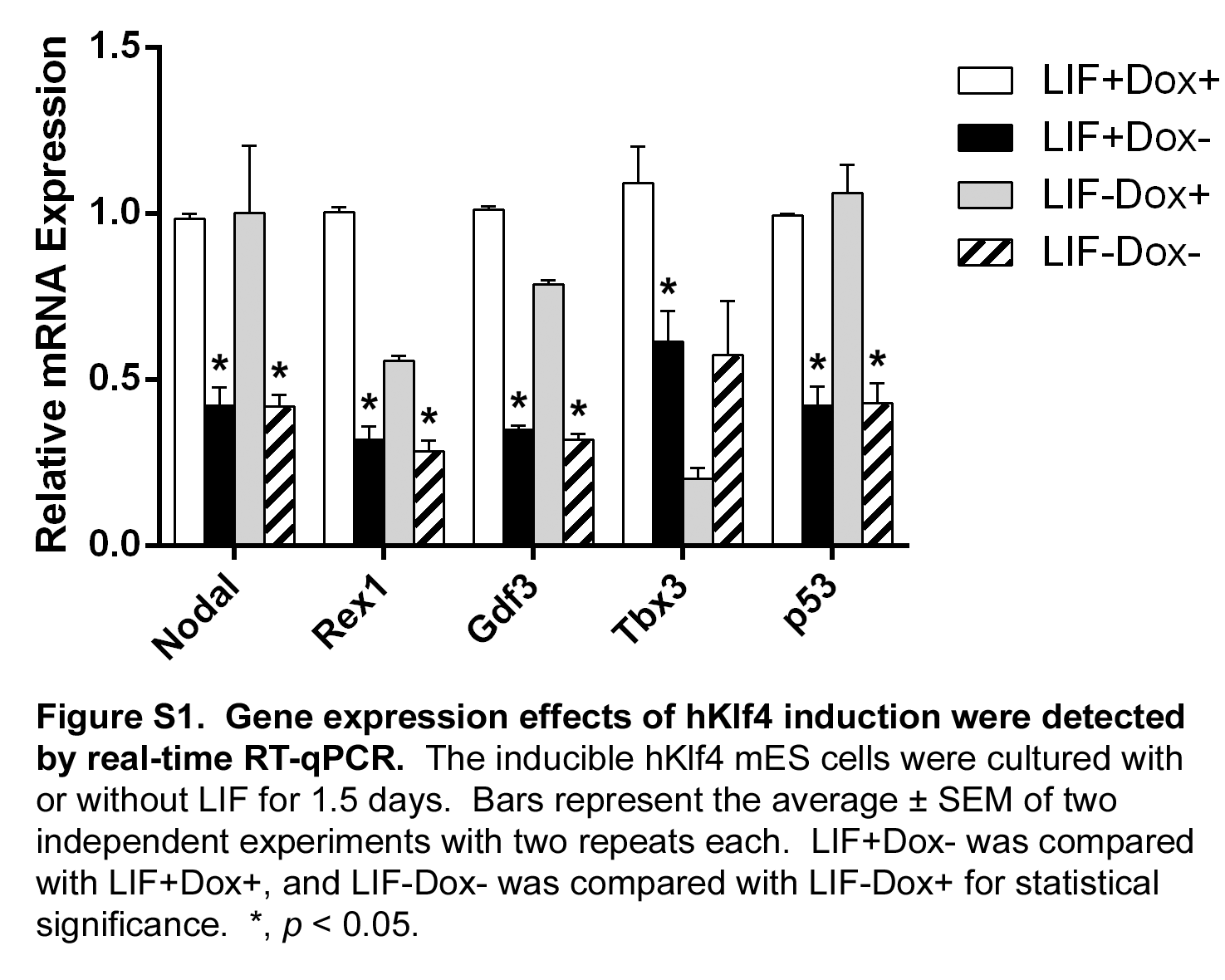

Supplement: Figure S1 — Gene expression effects of hKlf4 induction were detected by real-time RT-qPCR. (TIF) [file pone.0063532.s001.tif]
