# Supplementary material for: Regulatable In Vivo Biotinylation Expression System in Mouse Embryonic Stem Cells
Source: PLoS One. 2013 May 7;8(5):e63532. doi: 10.1371/journal.pone.0063532 (PMC3646753; doi:10.1371/journal.pone.0063532)
Supplement: Table S1 — Primers used for quantitative real-time PCR. (DOC) [file pone.0063532.s002.doc]

**Table S1. Primers used for quantitative real-time PCR**

|  | Forward Primer | Reverse Primer |
| --- | --- | --- |
| Transgene hKlf4 | ACGATCGTGGCCCCGGAAAAGGACC | ACCTGGAAAATGCTCGGTCGCATT |
| Total Klf4 | CAAGCCAAAGAGGGGAAGA | CGTCCCAGTCACAGTGGTAA |
| Endogenous mKlf4 | AGTGTGACAGGGCCTTTTCCAGGT | AAGCTGACTTGCTGGGAACTTGACC |
| Klf2 | CGGGCCTTCTCCAGGTCCGA | GAACCTGCCACAGTCGCGCA |
| Klf5 | GCATGGTGTGCCAACGCAGC | GCGCTCGCTCGCTCAGTTCT |
| Esrrβ | CAGGCAAGGATGACAGACG | GAGACAGCACGAAGGACTGC |
| Sox2 | GGTTACCTCTTCCTCCCACTCCAG | TCACATGTGCGACAGGGGCAG |
| Nanog | ATGCCTGCAGTTTTTCATCC | GAGGCAGGTCTTCAGAGGAA |
| Oct4 | GTTGGAGAAGGTGGAACCAA | CCAAGGTGATCCTCTTCTGC |
| Gdf3 | tgttcgtgggaacctgcttac | tctggagacaggagccatctt |
| Nodal | caagcctgttgggctctactc | cacgtccacatcttgcgcctg |
| Rex1 | CCGGGGAGAGGCGCTTTGTG | CCCGGCCTTTGCGTGGGTTA |
| Tbx3 | gtctcaggcctagaatccacag | aggctcccgaaaggcgacatag |
| p53 | TGTATCCCGAGTATCTGGAAG | GTCTTCCAGTGTGATGATGGTAA |
| β-actin | AACCCTAAGGCCAACCGTGAA | ACAGCCTGGATGGCTACGTA |
| Nanog enhancer | TGGGGTAAACTTAAGGCTATGG | AGCTCTAAGCCGGTTCTCATTT |
| Nanog upstream 3.3 kb region control | GCATAAACCTTGATATTTTGAACGGCCTATT | ACAGATGGACTAAAGCCCCTAAGTAGAAATCAT |
